# Sexing of Embryos at the Time of Twin Reduction: A Clinical Approach

**DOI:** 10.3390/ani13081326

**Published:** 2023-04-12

**Authors:** Fernando López-Gatius, Irina Garcia-Ispierto

**Affiliations:** 1 Agrotecnio Centre, 25198 Lleida, Spain; 2 Transfer in Bovine Reproduction SLu, 22300 Barbastro, Spain; 3 Department of Animal Science, University of Lleida, 25198 Lleida, Spain

**Keywords:** co-twin embryos, freemartinism, heterosexual twins, male embryo growth, sex differentiation, sexual organogenesis, twin growth patterns

## Abstract

**Simple Summary:**

In dairy cows, twin pregnancies and twinning are highly undesirable as they compromise health, welfare, and productive lifespans. We propose that the negative effects of twinning can be avoided by inducing twin reduction. Among dairy cows in their third lactation or more, the incidence of twin pregnancies may be as high as 30%, and different-sex twins represent around 50% of all twin pregnancies. This study provides new unique information on twin pairs during the late embryonic period (28–34 days of pregnancy) that allows for sex selection at the time of embryo reduction, such as differential ultrasound size measurements in heterosexual twins, different intrauterine embryonic growth patterns, and the different vulnerability of female and male embryos following the induced reduction in their co-twins. After heterosexual twin reduction, when the survival embryos were male, pregnancy loss was null.

**Abstract:**

This study provides new unique information on bovine twin pairs during the late embryonic period (28–34 days of pregnancy) in relation to (1) a predictive ultrasound measurement that was differential for sexing heterosexual twins; (2) intrauterine embryonic growth patterns in twin pairs; and (3) a higher vulnerability of female embryos compared to males following an induced embryo reduction in heterosexual twins. The study population comprised 92 dairy cows carrying bilateral twins. A length difference between co-twins equal to or greater than 25% in around 50% of pregnancies served to determine the sex of embryos with 100% accuracy in heterosexual twins, which was assessed four weeks later on the remaining fetus after twin reduction. The apparent rates of growth of twin pairs and of individual male and female embryos from day 28 to 34 of gestation were similar to established growth pattern standards for singletons. Mean embryo sizes in relation to gestational age were smaller by some 5 days’ growth equivalent in twins compared to singletons. After the reduction in the female embryo in heterosexual twins, the risk of male embryo loss was null. This new information allowed for sex selection at the time of twin reduction.

## 1. Introduction

In mammals, a male embryo develops faster than a female embryo before the differentiation of the gonads [1,2,3]. This phenomenon was already described in 1917 as the result of studying freemartinism in bovine twin pregnancies [4,5,6]. Subsequent work confirmed this finding [7,8,9]. Placental vascular anastomosis occurs in most twin pregnancies in cattle, and when heterosexual twins share the same circulation system, the female fetus becomes masculinized and, therefore, sterile [6,10,11]. Heterosexual twins account for some 50% of all twin pregnancies [12,13,14]. Twin pregnancies carry a greater risk of pregnancy loss than single pregnancies [15,16,17], and twin births lead to postpartum reproductive disorders [18,19,20,21], compromising the health, welfare, and productive lifespan of a cow. Consequently, twin pregnancies are detrimental to farming economics [22,23,24]. Multiple pregnancies, mostly twin pregnancies [25,26], may affect almost 30% (1827/6463) of cows in their third lactation or more [26,27]. Given that twin pregnancies are highly undesirable in dairy cattle, twin reduction by a manual rupture of the amniotic vesicle at the time of pregnancy diagnosis (28–34 days of gestation) is routine practice in commercial dairy herds under our surveillance [15,16]. Although this procedure is little used in dairy cattle, induced embryo reduction is a relatively common procedure in women [28,29,30] and mares [31,32,33]. 

Standards of late embryonic/early fetal growth and bovine conceptus viability have been extensively reported in ultrasound studies [34,35,36,37,38]. The embryonic period of gestation spans from conception to the completion of the stage of differentiation (approximately 45 days), while the fetal period runs from gestation day 45 until parturition [39]. Twin pregnancy is classed as bilateral (one fetus in each uterine horn, 44%) or unilateral (both fetuses in the same uterine horn, right or left, 56%) [26,27]. The likelihood of pregnancy loss may be up to five times higher for unilateral twins than for bilateral twins [40,41]. We designed the present study in bilateral twin pregnancies to examine the association between the co-twin-size differential at the time of induced embryo reduction (at 28–34 days of gestation) and fetal sex observed four weeks later. This new information could allow for sex selection at the time of twin reduction in identified heterosexual twin pregnancies. As the literature lacks embryo twin pair measurements during the late embryonic period in dairy cattle, a second objective was to explore the apparent rate of growth in individual twins. As a third objective, the possible effects of individual twin size, days of gestation, and embryo gender on heterosexual twin pregnancy loss rates were also examined.

## 2. Materials and Methods

### 2.1. Cows and Herd Management

This study was performed on three commercial Holstein-Friesian dairy herds kept on farms 1 km apart in north-eastern Spain (latitude 41.13 N, longitude −2.4 E). As individual twin measurements and the pregnancy loss rate was not significantly different among herds data, were grouped as derived from a single herd and “herd”, which were considered as a factor in binary logistic regression procedures. During the study period (October 2021 to January 2023), the mean number of lactating cows in the herds was 7330, and the mean annual milk production was 14,965 kg per cow. The mean annual culling rate was 30%. Cows were grouped according to age (primiparous plus secundiparous versus cows in their third lactation or more) and fed complete rations. The herds were maintained on a weekly reproductive health program in which pregnancy was diagnosed by transrectal ultrasonography from days 28 to 34 post-insemination using a portable B-mode ultrasound scanner equipped with a 5–10 MHz transducer (E.I. Medical IBEX; E.I. Medical Imaging, Loveland, CO, USA). Each ovary was scanned in several planes by moving the transducer along its surface to identify the ovarian structures, and the number and location of corpora lutea (CL) were recorded. The dorso/lateral surface of each uterine horn was scanned to detect the presence of twins. The pregnancy was checked again by ultrasound four weeks later. Pregnancy loss was recorded when this exam proved negative. The viability of an embryo/fetus was confirmed by the observation of a heartbeat in all exams. All gynecological exams and pregnancy diagnoses were performed by the same operator.

### 2.2. Experimental Design

**Figure 1:**
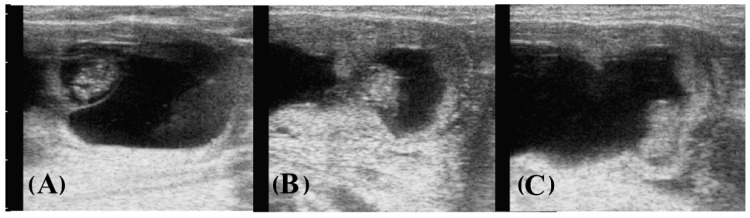
Sequence of gray-scale sonograms showing the twin reduction procedure on a day 29 twin embryo. Scale marks on the left are in cm. (**A**) The amniotic vesicle of the embryo is transrectally held with the thumb and half. (**B**) Pressure and rupture of the amniotic vesicle. (**C**) Disappearance of amnion and cessation of the embryonic heartbeat. Cows did not show any sign of discomfort.

Only cows in their third lactation or more carrying live bilateral twins (one embryo in each uterine horn) with two CL (one CL in each ovary) were included in this experiment. Primiparous and secundiparous cows were excluded because twins are much more common in cows in their third lactation or more [25,26,27], and the pregnancy loss rate is similar among cows with a parity of three or more [42,43]. The length of each embryo was measured on a frozen ultrasound frame using the electronic calipers of the ultrasound machine, and measurements were adjusted to the nearest one mm. As the embryo initially had the appearance of a straight line which developed a C shape on day 25 and an L shape on day 33 [35], the length of the long axis of the embryo visualized in a dorsoventral plane was recorded as the length of the embryo proper. For twins of different lengths, the length differential was defined as the percentage of difference with respect to the larger embryo. We assumed the larger embryo to be male and the smaller one to be female [1,2,3,37]. In these pregnancies, larger and smaller embryos were alternately ruptured on a weekly rotational basis, and fetal sex was determined from days 56 to 62 of gestation [44,45,46] in the pregnancy confirmation exam. Finding a size differential cutoff for the sexing of twins in the heterosexual group was the first objective of the present study. In the remaining pregnancies, in which a length that was differential between twins was not detected, the embryo located in the right uterine horn was ruptured. All cows received 1250 mg flunixin meglumine plus 100 µg GnRH immediately before or after reduction to promote the maintenance of gestation [15]. For induced twin reduction, the rupture of the amniotic vesicle of a twin embryo was guided by ultrasound and carried out by applying manual pressure. The amniotic vesicle was held and pressed with the thumb and half to cause a rupture (Figure 1). The time lapse between the vesicle subjection and the rupture was no longer than five seconds, and the cows did not show any sign of discomfort. Embryo death was assessed by the cessation of the heartbeat and was observed by ultrasound. All procedures of twin reduction were performed by the same operator.

In single pregnancies, factors such as parity (heifers versus parous cows), milk production, body condition score, circulating hormones and metabolites, insemination number, the sire of the embryo, and the horn of pregnancy could not be associated with embryo size during the late embryonic period [37,38]. Therefore, our second objective was to compare growth patterns in twins with established growth standards for singleton pregnancies. As embryo measurements were taken only once between 28 and 34 days of pregnancy, only apparent rates of growth for twin pairs from day 28 to 34 of the pregnancy and embryo measurements for each day of pregnancy were compared to the growth standards for singletons. Examining the possible effects of individual embryo measurements and days of gestation on pregnancy loss rates was this study’s third objective. Two unplanned appraisals were initiated during the course of this study. Once a clear cutoff was observed, which allowed for the sexing of twins), the apparent growth rate for female and male embryos was included in the second objective, whereas the embryo gender of the heterosexual twins was examined as part of the third objective.

In our geographical region of study, there are only two clearly differentiated weather periods: warm (May to September) and cool (October to April) [47,48]. The likelihood of pregnancy loss in twin pregnancies may be up to five times higher during the warm than cool period [49]. Temperatures for the study period were: 39 days of minimum temperatures < 0 °C and 7 days of maximum temperatures > 25 °C for October 2021 to April 2022 plus October 2022 to January 2023; and 0 days of minimum temperatures < 0 °C and 101 days of maximum temperatures > 25 °C for May to September 2022. To reduce the risk of pregnancy loss, cows were only included if they were healthy, as indicated by a body condition score of 2.5–3.5 on a scale from 1 to 5 [50], which produced more than 40 kg of milk per day, were free of clinical signs of disease during the study period (Days 28 to 62 of pregnancy), and became pregnant during the cool period of the year. Cows were included only once in the experiment. The final study population comprised 92 cows with twin pregnancies.

### 2.3. Data Collection and Statistical Analyses

The following data were recorded for each pregnancy: the day of pregnancy, length of individual twins, size differential between twins, semen-providing bull, pregnancy loss, and fetal sex in pregnancies with size differentials between twin pairs. Statistical analyses were performed using PASW Statistics 18 software (SPSS Inc., Chicago, IL, USA). Significance was set at *p* < 0.05. Variables are expressed as the mean ± standard deviation (S.D.).

As a result of the evident size differential cutoff predicting fetal sex, descriptive statistics were only used on these values. Embryo measurements and size differentials for each time point were assessed by one-way ANOVA. A correlation was performed between mean embryo size values and the day of pregnancy. The relative contributions of each factor to the probability of pregnancy loss were determined by binary logistic regression. Pregnancy loss was considered to be the dependent variable, and the day of pregnancy (class variable: days 28, 29, 30, 31, 32, 33, and 34), the mean length of twin pairs, and the size differential between co-twins (continuous variables), and the sex of the embryo, coded as a class variable (non-sexed, female, and male), were considered factors in the analysis. The semen-providing bull (sire of the embryo in this study), a main factor influencing pregnancy maintenance independent of its fertilizing capacity [51,52,53], was not included in the model because a large number of sires was used (14 sires). Four sires provided semen for a single pregnancy each, with six sires for two pregnancies and four sires for three or more pregnancies. Any effect on the semen-providing bull was assumed to be randomly distributed and a possible component of error in the experiment. Regression analyses were conducted according to the method of Hosmer and Lemeshow [54]. Basically, this method consisted of five steps as follows: the preliminary screening of all variables for univariate associations; the construction of a full model using all the significant variables arising from the univariate analysis; a stepwise removal of non-significant variables from the full model and comparison of the reduced model with the previous model for model fit and confounding; an evaluation of the plausible interactions among variables; and assessment of model fit using Hosmer-Lemeshow statistics. Variables with univariate associations showing *p* values < 0.25 were included in the initial model. Model reduction continued until only significant terms according to the Wald statistic remained in the model at *p* < 0.05.

## 3. Results

A length differential was not observed in 34 (37%) of the 92 twin pairs. Table 1 shows length differentials on days 28–34 of pregnancy, which was used to determine embryo sex. 

Descriptions of embryo measurements (mean, S.D., minimum and maximum) and length differentials between male and female embryos are provided in Table 2. Sex was assessed in 45 co-twins (48.9%) which showed length differentials equal to or higher than 25% (Table 1 and Table 2). Length differentials from 8% to 15% detected in the remaining 13 twin pregnancies (14.1%) were not associated with subsequently determined fetal sex (Table 1). The cutoff for embryonic sex determination was set at a length differential equal to or greater than 25%. This meant that embryo sex could be determined in 48.9% (90 co-twins) of twin pregnancies during the late embryonic period (28–34 days of pregnancy) based on the embryo length differential. 

**Figure 2:**
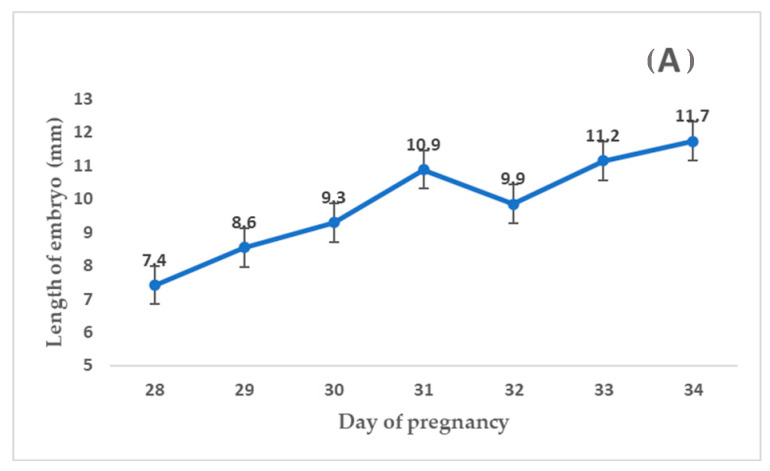

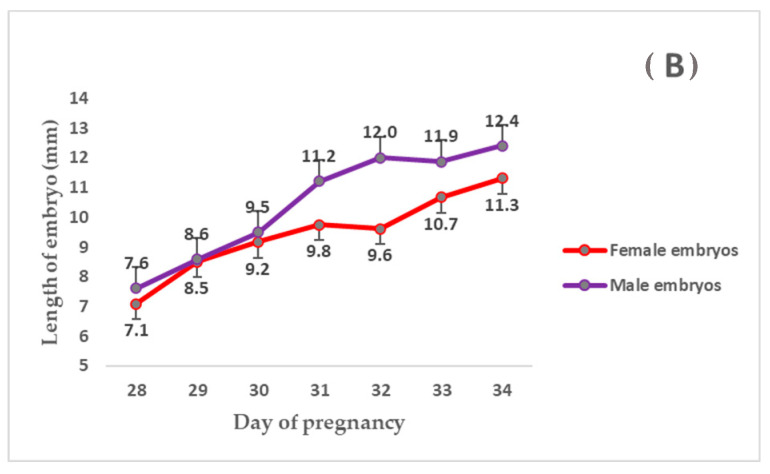
Mean lengths (±S.D.) for the total sample of co-twins. (**A**) *n* = 184 for male and female embryos. (**B**) *n* = 90 from day 28 to 34 of pregnancy.

Figure 2 shows the embryo size increases produced over time. A significant positive correlation was observed both for the total sample of twins and for male and female embryos (*p* < 0.0001). The mean percentage of the difference between co-twins with respect to the larger embryo (length differentials) was similar for each day of pregnancy.

Of the 92 cows enrolled in this study, 23 (25%) experienced pregnancy loss after twin reduction. Logistic regression analysis indicated no significant effects in terms of the day of pregnancy, the mean length of twin pairs, and the size differential between co-twins on pregnancy loss. Embryo sex was the only variable selected by the logistic procedure (Table 3). Taking non-sexed twin pairs as a reference, male embryos gave rise to an incidence of pregnancy loss that was significantly (*p* = 0.01) reduced by a factor of 0.08. As these results were unexpected, two further regression analyses were performed. First, when male embryos were used as the reference sex of the embryo variable, results were similar, and the incidence of pregnancy loss for female embryos was increased by a factor of 13. Finally, after removing the variable sex of the embryo, the length differential was the only variable remaining in the model, and one unit increase in the length differential significantly reduced the incidence of pregnancy loss by a factor of 0.92 (95% confidence interval: 0.86–0.97; *p* = 0.005).

## 4. Discussion

This study provides new and unique information on bovine twin pairs during the late embryonic period indicating that a predictive threshold value using ultrasound size differential measurements between co-twins can be used to determine the sex of heterosexual twins (50% of all twin pregnancies in cattle [12,13,14]); twin pairs showed some distinguishing intrauterine embryonic growth pattern characteristics compared to established standard singletons; and female embryos showed a greater vulnerability than their male partners following induced embryo reduction.

In the present study, length differentials between co-twins equal to or greater than 25% proved valuable to predict the sex of embryos at pregnancy diagnosis (28–34 days of pregnancy) with an accuracy of 100% for fetal sex, determined four weeks later on the remaining fetus after twin reduction. In contrast, length differentials from 8% to 15% were not useful. In effect, based on differentials in this last range for twin pairs, only five out of a total of twelve fetuses were of the expected sex. In other words, these were homosexual twin pregnancies. In an extensive study, sex ratios for twin pairs were 24.9% (male/male), 48.7% (male/female), and 26.4% (female/female) [12]. Probably, homosexual twins showed small growth differences. The fact that no size differentials between 15% and 25% were observed reinforces this statement. Our study population consisted of cows with two CLs suggesting that most of the homosexual twins were dizygotic. This means independent double ovulation and independent embryogenesis. In fact, dizygotic twins have the same genetic relationship as ordinary brothers or sisters, which may be the reason for small growth differences.

While we did not take measurements over time on the same co-twins, apparent rates of growth in twin pairs and of individual male and female embryos from day 28 to 34 of gestation resembled the average growth pattern observed in singletons [34,35,36,37,38]. When taking Curran’s work as a reference [35], however, the mean embryo size in relation to the age of gestation was smaller by about 5 days’ growth, which was equivalent in twins compared to singletons. For example, the mean lengths of a singleton on days 28 and 34 were reported at 9.5 and 16.5 mm, respectively [35], while means of 7.4 and 11.7 mm were observed here on the same days of pregnancy for twin pairs. On days 22 and 29 of pregnancy, singletons were 6 and 12 mm long [35]. This could suggest that, for any gestational age, twins are younger than singletons. The truth is that twin pregnancies reach parturition up to eight days earlier than single pregnancies [55,56], and although smaller calves are delivered, twin newborns show the same viability as singletons [56]. The smaller size of twins may be the result of competition for resources in the uterine environment and placental system.

In our study, efforts were made to reduce the risk factors associated with pregnancy loss. Thus, we only included cows in their third lactation or more with a similar risk of loss [42,43] that became pregnant during the cool period of the year, when in our region, there was a very low risk of twin pregnancy loss [49]. In this way, we tried to examine the possible effect of the day of pregnancy and embryonic measurements on the risk of loss. A reduced embryo size could not be associated with pregnancy loss contrasting with the situation for single pregnancies, in which a small embryo was considered a predictor of pregnancy loss [57,58]. Only embryo sex was a factor included in the logistic regression. Unexpectedly, female embryos appeared to be adversely affected by the rupture of their male co-twin. Conversely, after a reduction in the female embryo, the risk of male embryo loss was greatly reduced by a factor of 0.08 (0% under our work conditions). In humans, pregnancy failure is more likely if the baby is a boy [59,60,61,62]. Boys grow faster than girls before implantation, and this makes them more vulnerable to compromised nutrition [63,64,65]. Probably, in all mammals, life in the maternal uterus is more dangerous for male embryos than for female embryos. The widely reported size difference in favor of the male embryo [1,2,3,4,5,6,7,8] may support this notion. The present results could also confirm the idea that male embryos are more resistant only in the case of heterosexual twins.

The pregnancy loss rate was similar for unsexed twins (34%) and female embryos (30.4%), being slightly higher than the 25% described after induced embryo reduction in bilateral twin pregnancies [15,16]. Probably, losses for heterosexual twins have led to reduced reported incidences of pregnancy loss in previous studies. Total losses were also 25% (23/92) in the present study. The question that arises is why losses for male/male homosexual twins, accounting for 50% of all homosexual twin pregnancies [12], did not reduce the loss rate recorded in the set of unsexed twins. Could this be due to an XX/XY chimerism in heterosexual twins in addition to the more advanced development of the male compared to female co-twin, which would favor the maternal–embryo relationship and so the maintenance of gestation in the male? Bovine freemartinism likely remains the best-known rather than best-understood example of abnormal sexual differentiation in mammals [13,14].

Although XX/XY chimeras have been described in both bull and heifer calves from single births [66,67,68], the single-born freemartin remains unknown [14,69]. Determining the level of XX/XY chimerism in males born after a reduction in their female co-twins may help clarify this point.

Embryo sex selection at the time of twin reduction in cows in their third lactation or more has acquired particular relevance. The use of sex-sorted semen has increased enormously over the past two decades, particularly in heifers and primiparous cows [70,71,72]. As a result, producers generate their own replacement females from a smaller group of cows, leading to the massive use of conventional beef semen in dairy herds, particularly in multiparous cows [72,73]. In this situation, maintaining the male embryo partner in heterosexual twin pregnancies should maximize the market value of cross-bred calves after birth.

## 5. Conclusions

The length differentials between co-twins equal to or greater than 25% detected in around 50% of twin pregnancies proved valuable to determining the sex of embryos with 100% accuracy and assessed four weeks later on the remaining fetus after twin reduction. Apparent rates of growth for the twin pairs and of individual male and female embryos from day 28 to 34 of gestation were similar to growth standards for singletons. Mean embryo sizes in relation to gestational age were smaller by about 5 days’ growth equivalent in twins than established singleton standards. After heterosexual twin reduction, when survival embryos were male, pregnancy loss was null. This new information allows for sex selection at the time of heterosexual twin reduction in dairy cattle.

## Figures and Tables

**Table 1:** Threshold length differentials on day 28–34 of pregnancy, defined as the percentage of difference between co-twins with respect to the larger embryo, used to determine embryo sex (*n* = 92 twin embryo pairs).

Length Differential	Incidence	Pregnancy Loss ^(a)^	Expected Fetal Sex ^(b)^
0%	34 (37%)	15 (44.1%)	Undetermined
8–11%	6 (6.5%)	0 (0%)	2 (33.3%)
12–15%	7 (7.6%)	1 (14.3%)	3 (50%)
≥25%	45 (48.9%)	7 (15.6%)	38 (100%)

^(a)^ On day 56–62 of pregnancy. ^(b)^ In cows still pregnant.

**Table 2:** Mean, standard deviation (S.D.), minimum, and maximum values of measurements for the total number of embryos and sexed embryos recorded from days 28 to 34 of gestation (*n* = 92 twin embryo pairs).

Embryo Measurements	*N*	Mean	S.D.	Minimum	Maximum
Length (mm)					
Total no. embryos	184	9.4	2.5	5	15
Undifferentiated co-twins	94	9.4	2.1	5	13
Males	45	11.6	2.3	8	15
Females	45	7.2	1.3	5	10
Length differential (%) ^(a)^					
Male versus female embryos	45	35.5	18.5	25	50

^(a)^ Percentage difference between twin pairs with respect to the larger embryo.

**Table 3:** Odds ratios of the pregnancy loss rate variables included in the final logistic regression model (*n* = 92 twin pregnancies).

Factor	*n*	Pregnancy Loss	Odds Ratio	95% Confidence Interval	*p*
Non-sexed embryos	16/47	34%	Reference		
Female embryos	7/23	30.4%	0.8	0.7–1.3	0.91
Male embryos	0/22	0%	0.08	0.01–0.62	0.01

R^2^ Nagelkerke = 0.22.

## Data Availability

The data presented in this study are available on request. These data are not publicly available to preserve the data privacy of the commercial farms.
